# On the Origin of Solid Bodies in Synovial Cavities

**Published:** 1847-09

**Authors:** 


*On the Origin of Solid Bodies in Synovial Cavities.* By Dr. BIDDER.—The author had charge of a case of long standing swelling of the knee joint, which finally opened, allowing a quantity of granular matter to escape. The grains composing this matter were of a uniform size,—$1\frac{1}{2}'''$ long, $\frac{3}{4}'''$ broad, $8\frac{1}{2}'''$ thick,—regular, flat-oval, clumped together in masses of variable size by a glutinous transparent fluid, present in but a very small quantity; they were highly elastic, presented no trace of a pedicle; their cut surface seemed homogeneous to the naked eye, and presented no trace of organization under the microscope; a chemical examination showed them to be composed of albumen. Mickel's opinion, so recently substantiated by Hyrtl, does not, therefore, hold good in every case; our author acknowledging its correctness in many cases, as well as the possibility of some cases arising from hydatids, (Dupuytren,) viz. those in which the bodies are possessed of a laminated structure, and have an internal cavity, notwithstanding that other distinctive marks may be lost, (vide Gluge. Anat. Mic.,) throws out another hypothesis as an explanation of their mode of origin in cases like the present, viz., that in certain cases an increased flow of blood, and consequent secretion of synovia, may force off the epithelium cells; that these subsequently increase, partly by endosmosis, partly by precipitation on their external walls; the peculiar life of the cell wall in certain cases altering the contents both with respect to colour and consistency, no membrane being perceptible under the microscope, may proceed from its stretched and thinned condition, from its being originally structureless, or from its homologation with its contents. The bodies examined by him consisted almost entirely of albumen, easily obtained from the synovia: their uniform size likewise presupposed their origin to have been from similar forms, endowed with similar capacities for life, conditions fulfilled by the epithelium cells. This theory can only hold good where the synovial cavities have an epithelial covering, which is wanting in bursæ mucosæ, and mucous sheaths of tendons.—*Ibid, from Ibid.*

